# A Multimodal Patient-Centered Teleprehabilitation Approach for Patients Undergoing Surgery for Breast Cancer: A Clinical Perspective

**DOI:** 10.3390/jcm13237393

**Published:** 2024-12-04

**Authors:** Kenza Mostaqim, Astrid Lahousse, Simone Ubaghs, Annick Timmermans, Tom Deliens, Marian Vanhoeij, Christel Fontaine, Eric de Jonge, Jan Van Hoecke, Laura Polastro, Michel Lamotte, Antonio Ignacio Cuesta-Vargas, Eva Huysmans, Jo Nijs

**Affiliations:** 1 Pain in Motion Research Group (PAIN), Department of Physiotherapy, Human Physiology and Anatomy, Faculty of Physical Education and Physiotherapy, Vrije Universiteit Brussel, Laarbeeklaan 103, 1090 Brussels, Belgiumsimone.ubaghs@vub.be (S.U.); eva.huysmans@vub.be (E.H.); 2 Research Foundation—Flanders (FWO), Leuvensesteenweg 38, 1000 Brussels, Belgium; 3 REVAL Research, Faculty of Rehabilitation Sciences, Universiteit Hasselt, Agoralaan, 3590 Diepenbeek, Belgium; annick.timmermans@uhasselt.be; 4 Department of Physical Medicine and Physiotherapy, University Hospital Brussels, 1090 Brussels, Belgium; 5 Movement and Nutrition for Health and Performance (MOVE) Research Group, Department of Movement and Sport Sciences, Vrije Universiteit Brussel, Pleinlaan 2, 1050 Brussels, Belgium; tom.deliens@vub.be; 6 Department of Surgical Oncology, University Hospital Brussels, 1090 Brussels, Belgium; marian.vanhoeij@uzbrussel.be; 7 Department of Medical Oncology, University Hospital Brussels, 1090 Brussels, Belgium; christel.fontaine@uzbrussel.be; 8 Department of Gynecology, Ziekenhuis Oost-Limburg, 3600 Genk, Belgium; 9 Department of Physiotherapy, Ziekenhuis Oost-Limburg, 3600 Genk, Belgium; jan.vanhoecke@zol.be; 10 Department of Medical Oncology, Institut Jules Bordet, Hopital Universitaire de Bruxelles HUB, 1070 Brussels, Belgium; laura.polastro@hubruxelles.be; 11 Department of Physiotherapy, Hopital Erasme, 1070 Brussels, Belgium; michel.lamotte@hubruxelles.be; 12 Clinimetria Research Group, Department of Physiotherapy, Faculty of Health Sciences, Universidad de Malaga, 29071 Malaga, Spain; acuesta@uma.es; 13 Unit of Physiotherapy, Department of Health and Rehabilitation, Institute of Neuroscience and Physiology, Sahlgrenska Academy, University of Gothenburg, 405 30 Gothenburg, Sweden

**Keywords:** breast cancer, prehabilitation, patient-centered care, telecommunication

## Abstract

Breast cancer is the most common malignancy among women worldwide, and advances in early detection and treatment have significantly increased survival rates. However, people living beyond breast cancer often suffer from late sequelae, negatively impacting their quality of life. Prehabilitation, focusing on the period prior to surgery, is a unique opportunity to enhance oncology care by preparing patients for the upcoming oncological treatment and rehabilitation. This article provides a clinical perspective on a patient-centered teleprehabilitation program tailored to individuals undergoing primary breast cancer surgery. The proposed multimodal program includes three key components: patient education, stress management, and physical activity promotion. Additionally, motivational interviewing is used to tailor counseling to individual needs. The proposed approach aims to bridge the gap between diagnosis and oncological treatment and provides a holistic preparation for surgery and postoperative rehabilitation in breast cancer patients. The aim of this preparation pertains to improving mental and physical resilience. By integrating current evidence and patient-centered practices, this article highlights the potential for teleprehabilitation to transform clinical care for breast cancer patients, addressing both logistical challenges and holistic well-being.

## 1. Introduction

The latest GLOBOCAN report (2022) [1] estimated that 3 out of 10 global premature deaths from noncommunicable diseases are caused by cancer. In women, breast cancer (BC) is the most prevalent malignancy diagnosed worldwide with over 2.3 million new cases per year (>11 k in Belgium [2]), comprising 11.6% of all newly diagnosed cancer cases [1]. Especially in high-income countries, incidence rates have been increasing over time, with the 5-year survival rate exceeding 90%, mainly due to improved, early detection through mammographic screening [1]. Luckily, early detection together with innovation and improvement in treatment also resulted in relatively decreased mortality rates [1,3,4,5]. Consequently, annually, more patients with BC transition to the survival stage, where the fortune of surviving is often overshadowed by late consequences from cancer treatment such as pain and disability, but also anxiety, depression, and fear of cancer recurrence [3,4,6]. These late consequences not only significantly impact the patient’s quality of life but also lead to an important long-term socioeconomic burden and a need for supportive care [6,7,8]. 

Curative treatment for BC is tailored to each patient based on their diagnosis, prognosis, and specific characteristics [7]. Typically, these approaches involve a combination of medical interventions, such as surgery, radiation therapy, chemotherapy, targeted therapy, and/or endocrine therapy [7]. Surgical removal of the primary breast tumor is a cornerstone for disease management in the majority of BC patients (90% of individuals diagnosed with stage I-III BC receives surgery [9]) [10,11]. 

Following curative treatment, BC rehabilitation is an evidence-based and well-integrated aspect of the oncological care pathway [12,13,14]. However, patient adherence is often low due to cognitive-emotional barriers and the lack of a behaviorally informed approach, which leads to limited treatment success. Furthermore, current BC rehabilitation programs mainly focus on improving upper limb function, mobility, and prevention/treatment of lymphedema. While these are important, they insufficiently address other critical aspects of recovery, such as supporting patients in adopting or maintaining a healthy, active lifestyle or addressing their psychological and/or emotional needs [3,4,6,7]. Indeed, qualitative studies reveal that BC patients/survivors experience an unmet need for supportive care in areas like emotional support, psychological coping, and behavior change among breast cancer survivors [3,4]. Despite these reported gaps, clinical practice guidelines emphasize the crucial role of supportive care for both mental and physical well-being, as well as the promotion of a healthy, active lifestyle that extends beyond more upper limb function, influencing symptoms and even cancer recurrence [15,16,17,18]. This mismatch between patients’ needs and the focus of current rehabilitation programs underscores the urgency for more comprehensive, behaviorally informed approaches that address both physical and psychosocial dimensions of recovery. 

Interestingly, the timeframe following BC diagnosis and surrounding curative treatment represents a teachable moment in life [5,6,7,19]. As such, it provides an important window of opportunity for interventions to optimally guide patients throughout their cancer care trajectory and to prepare them for an active healthy life after cancer, including providing them with the necessary cues to initiate a sustainable behavioral change [19]. This aligns with the goals of prehabilitation, which aims to improve factors related to the period around surgery to enhance the patient’s physical and mental capabilities and reduce the immediate and long-term side effects of cancer treatment [7,20]. The concept of “prehabilitation” is gaining noteworthy acceptance in the field of oncology [7,21], as it might lead to improved clinical, psychological, physical, and quality-of-life outcomes [6]. Promising results of prehabilitation have been found in several surgical populations, including oncological patients [5,6]. Additionally, Scheede-Bergdahl et al. state that the presurgical period is an ideal period to optimize the physical and emotional status of the patient before the stress of the surgery and could be key to addressing patients’ individual needs [22,23]. To be able to address these complex needs, a shift from single-mode to multimodal biopsychosocial approaches is needed, which is in line with the current movement towards integrative oncology [6,7,10,24,25]. On the other hand, the care pathway between BC diagnosis and surgery can be challenging and overwhelming to patients and is kept as short as possible to achieve the best chance of long-term survival [26,27]. Therefore, the format and components of a perioperative intervention should be carefully chosen to ensure feasibility while maintaining quality of care and accommodating the complex physical and emotional needs of BC patients at the beginning of their care trajectory [6,7,19]. 

Here, a clinical perspective on multimodal patient-centered teleprehabilitation for patients undergoing surgery for BC considering the current evidence, patient preferences, and logistical challenges specific to the care pathways is offered.

## 2. Teleprehabilitation for Patients Undergoing Breast Cancer Surgery: Application in Clinical Practice

### 2.1. Target Population

Although the application could be extended to BC patients following a different care trajectory, the proposed prehabilitation intervention was designed to fit in the care pathway of BC patients receiving surgery as a primary curative treatment. Acknowledging the physically and emotionally challenging nature of breast surgery and the potential for functional impairments and diminished well-being, the proposed prehabilitation intervention is individually tailored [4,10,23,28,29]. 

### 2.2. Tailoring Multimodal Patient-Centered Teleprehabilitation for Patients Undergoing BC Surgery

**Figure 1:**
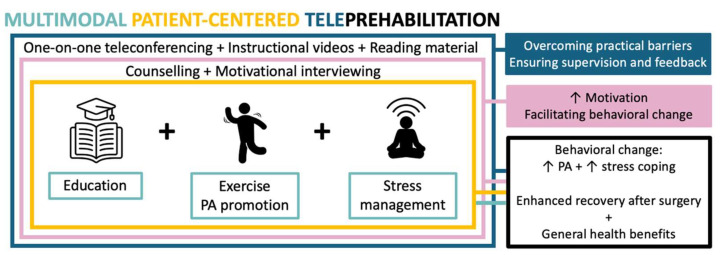
Rationale for multimodal patient-centered teleprehabilitation in patients with breast cancer. Legend: PA—physical activity. The arrows stand for “more”.

We propose a multimodal patient-centered teleprehabilitation program containing patient education, stress management, and physical activity promotion with integration of counseling guided by motivational interviewing throughout. The key reasons for this approach are outlined below and summarized in Figure 1. 

Based on the available literature, most prehabilitation interventions for patients with BC are focusing on exercise therapy and/or physical activity [5,6]. This is not surprising as strong evidence supports an inverse relationship between physical activity and cancer recurrence, (all-cause) mortality, and persistent symptoms after BC [1,6,19,30,31,32]. Additionally, engaging in physical activity following BC diagnosis and during treatment has been associated with improved psychological preparedness for surgery and treatment, lower anxiety and distress, and improved quality of life [5,19,33]. These findings have led to the extension of the general World Health Organization’s (WHO) physical activity guidelines (performing at least 150 min of moderate physical activity/week) to patients living with and beyond BC [6,17,19]. Despite these biomedical and psychosocial advantages, physical activity levels of patients with BC remain generally low, and single-mode exercise prehabilitation interventions only show limited outcomes [6,19]. This is believed to be due to the fact that these interventions fail to accommodate cognitive-emotional and/or practical barriers and are insufficiently behaviorally informed to induce a sustainable change towards an active healthy lifestyle [6,7,19,23].

In this sense, it needs to be considered that to increase the patient’s intrinsic motivation to adhere to an intervention, they should understand why their engagement can be helpful and how it will support their recovery and continued life [34]. This can be achieved by integrating a tailored education component into perioperative management to prepare the patient for cancer treatment, support the active therapy components (including addressing barriers for physical activity), and address any unhelpful cognitive-emotional factors [35,36,37,38,39,40,41,42,43]. Importantly, unhelpful cognitive-emotional factors are not only barriers to active treatment adherence but are also known risk factors for unfavorable outcomes after surgery [36,37,38,39,40,41,42]. Qualitative research underlined that patients with BC value educational components of prehabilitation, which provides them with a sense of self-control in a period where it seems that many things are out of their own control [44].

To further support the emotional needs of patients with BC, it has been suggested to add stress management to their perioperative care trajectory [6,7,44]. Receiving a BC diagnosis can naturally affect psychological well-being, with most patients experiencing anxiety symptoms, feelings of loss of control, and fear for the future, resulting in overall psychological distress [3,4,7]. These cognitive-emotional consequences can hamper the patient’s ability to process information and initiate a behavioral change (e.g., increasing physical activity levels) [6,19]. As such, providing stress management early in the care trajectory of patients with BC can not only support them in coping with their emotional concerns but it may also reduce barriers to adopt a healthy active lifestyle [7,45]. 

Furthermore, it is crucial to understand how best to communicate throughout an intervention that is aiming for a behavioral change. Motivational interviewing is a directive, collaborative, patient-centered communication approach for eliciting and enhancing motivation for behavior change by helping patients to resolve ambivalence (e.g., “Why would I invest in exercises while I’m busy surviving?”) and uncertainty [46,47,48,49]. Accounting for individual patient perceptions by addressing them through motivational interviewing holds the potential for a patient-centered approach that boosts engagement for behavioral change and treatment adherence [48,49,50]. Moreover, unhelpful disease perceptions affect quality of life after oncologic treatment, and modifying these perceptions early is therefore a potential strategy to create an optimal baseline for BC treatment [50]. By integrating motivational interviewing in prehabilitation for patients with BC, the teachable moment surrounding diagnosis and treatment can optimally be used to guide patients out of their own intrinsic motivation and empowering experience towards an active healthy lifestyle during and after cancer treatment.

Next to the patients’ clinical needs, also practical barriers to participation in and adherence to a perioperative intervention need to be countered [7,19,34,51,52]. Considering transportation and time issues [10], patients with cancer prefer home-based exercise programs [51]. This calls for interventions that use telecommunication to overcome these barriers without unnecessarily increasing healthcare costs (e.g., for home visits) while ensuring supervision and/or guidance from a therapist. Prior to the COVID-19 pandemic, telehealth was rarely utilized in oncological care [53], but since then, telerehabilitation services have developed rapidly. Patients with BC value their benefits such as reduced traveling barriers, flexible treatment hours, increased access to care, and reduced socioeconomic barriers to engage in the interventions [53,54,55,56]. On the other hand, telerehabilitation requires a certain level of digital literacy, which might be challenging for some. However, a recent feasibility study in patients with cancer showed that people who experience a self-perceived lack of digital ability can perceive participation in a telerehabilitation program as an opportunity to upskill [56].

Multimodal lifestyle-oriented prehabilitation has been found to be feasible and can lead to improved health outcomes in patients with BC [10,44,56,57]. However, due to practical barriers, Wu et al. (2021) advised setting up teleprehabilitation programs [10]. In response to this, they explored the feasibility of a multimodal teleprehabilitation intervention in patients with BC and concluded that such an intervention was equally feasible and could favorably affect self-perceived health and fatigue [56]. Additionally, they identified patient-specific facilitators for teleprehabilitation adherence: integration of wearables for self-monitoring, one-on-one therapist contacts, instructional videos supporting home-based exercises, and the fact that they could exercise in their safe home environment instead of in front of other people [56]. Furthermore, pilot/feasibility trials confirmed that the preoperative timeframe might be too short in patients with BC undergoing primary surgery to achieve a sustainable lifestyle change, indicating that a more longitudinal approach of (p)rehabilitation is needed in this population [10,57]. 

**Figure 2:**
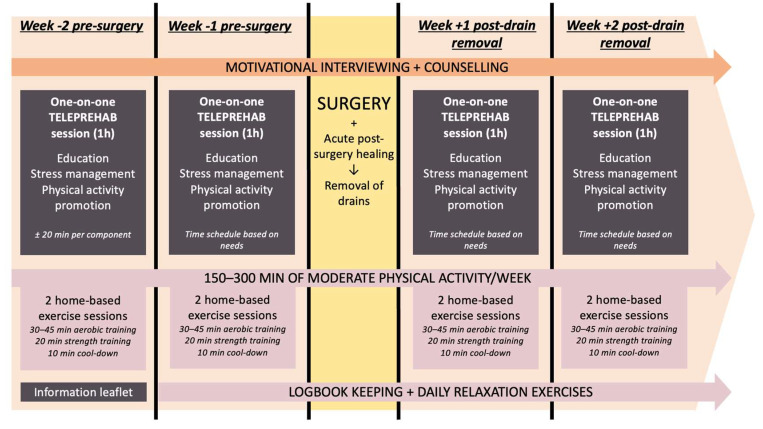
Overview of the different components of the multimodal teleprehabilitation intervention for patients with BC.

Taken together, considering the clinical needs of the BC patient, the logistical challenges, the current state-of-the-art, and our clinical expertise, we propose a treatment protocol with four one-on-one patient-centered teleprehabilitation sessions (two pre- and two post-surgery), each lasting approximately one hour. These are supplemented by an informational booklet and home-based exercises (i.e., exercise sessions and relaxation exercises) at home. The format of the proposed prehabilitation intervention, including the different components, are summarized in Figure 2. 

### 2.3. Format and Content of the Proposed Multimodal Patient-Centered Teleprehabilitation for Patients Undergoing BC Surgery

#### 2.3.1. One-On-One Sessions and Home-Based Exercises

The proposed format contains four one-on-one sessions between the patient and the physical therapist delivered via teleconferencing. Each one-on-one session covers the three main components: education, stress management, and physical activity promotion with an overarching goal to mentally and physically prepare individuals for their BC treatment and life thereafter, including utilizing the teachable moment surrounding cancer diagnosis and treatment to adopt a healthy active lifestyle [6,7]. The first one-on-one session, two weeks prior to surgery, focuses on education, stress management strategies, and physical activity promotion. Equal time (approximately 20 min each) is initially allocated to each of the components to provide a balanced foundation (Figure 2). The second one-on-one session, one week prior to surgery, allows more flexibility in time allocation, prioritizing the components where the patient needs the most support, while still covering all three aspects. In the 2 weeks post-drain removal, two additional sessions are conducted weekly. These sessions continue to provide tailored support for each of the components as the patient recovers post-surgery, the allocation of time during the sessions shifting based on individual patient needs. For example, a patient experiencing high levels of anxiety may require more focus on stress management, while another struggling with physical activity adherence may benefit from additional exercise guidance.

The one-on-one sessions can be guided by motivational interviewing as a directive, collaborative, patient-centered communication approach to elicit and enhance motivation for behavior change by helping patients to resolve ambivalence and uncertainty [46,47]. Accounting for individual patient perceptions [50] by addressing them through motivational interviewing holds the potential for a patient-centered prehabilitation approach to boost engagement and treatment adherence [49]. After all, disease perceptions affect the quality of life after oncologic treatment, and influencing patients’ disease perceptions is therefore a potential strategy to create an optimal baseline for BC treatment [50].

The use of motivational interviewing aims to develop autonomous motivation by increasing perceived competence and self-regulation [58]. It implies that components of the prehabilitation intervention (i.e., education, stress management, and exercise therapy) are proposed based on a guided question-and-answer play between the patient and the therapist. Motivational interviewing implies that the therapist is supportive, empathetic, positive, and hopeful, and it relies on the therapeutic alliance to assist in changing certain health behaviors based on the patient’s internal thoughts. Motivational interviewing also aims to strengthen personal commitment by respecting the individual’s autonomy and assists them in reaching a specific goal by exploring personal intentions or reasons for change [46,47]. Hence, the focus of the teleprehabilitation intervention differs based on the preferences, attitudes, illness perceptions, and self-efficacy of the patient. During each session, the patient’s actions regarding the teleprehabilitation intervention can be evaluated, discussed, and reinforced or tailored in more detail. To achieve this, patients can hold a logbook of their therapy progress in which they can log their home-exercise sessions and any concerns or uncertainties. On the one hand, this logbook can serve as a self-monitoring tool, and on the other hand, it can guide the feedback moment at the beginning of each one-on-one session where the logbook can be reviewed and discussed, including resolving any uncertainties.

**Component 1**. Education

The education component of BC prehabilitation is a crucial element aimed at empowering patients with the necessary knowledge and skills to actively engage in their well-being throughout the treatment process. The education should overarch all intervention components, including the following topics: (1) the consequences of stress and low physical activity levels; (2) encouragement and examples of how improving stress tolerance and increasing physical activity can influence not only quality of life but also the oncological treatment [59,60]; (3) the influence of (unhelpful) cognitive-emotional factors on surgical outcome, but also strategically discussing their role as a barrier for treatment participation [39,40,41,42].

Regarding physical activity, the education aims at increasing the patient’s understanding of the importance of an active lifestyle and reshaping any unhelpful perceptions and cognitions that form a barrier for physical activity participation [43,61,62]. To achieve this, the following knowledge can be transferred to the patient in an interactive way and with integration of patient-specific examples and experiences. First of all, engaging in active lifestyle promotion can improve quality of life but also hold promise for reducing cancer-related side effects and comorbidities in cancer survivors [61,63], including enhancements in physical functioning and quality of life that persist for several months after treatment [64]. Second, physical activity holds an inverse relationship with all-cause and BC-related mortality, as well as BC recurrence [65,66]. For instance, the cohort study by Chen et al. suggests that BC survivors who were active or moderately active had a 60% lower risk of death compared to those who were insufficiently active [67], underscoring the significance and value of adopting a physically active lifestyle if one is not already. Additionally, physical inactivity increases the risk of recurrence and/or the development of other chronic diseases in BC survivors [68,69]. Third, moderate physical activity has a positive impact on sequelae of cancer treatment, including cancer-related fatigue, one of the most debilitating symptoms in cancer patients and survivors [70,71]. A significant reduction in cancer-related fatigue is seen in women who remain lightly to moderately physically active throughout their BC treatment [64,72]. Even after completing cancer treatment, exercise has positive effects on reducing cancer-related fatigue, depression, anxiety, and stress [73]. Finally, sedentarism could be addressed. With the link between a sedentary lifestyle and the occurrence of cancer being widely recognized, even after being diagnosed with BC, breaking up sedentary time appears to reduce the risk of cancer mortality [74]. 

In other words, after this part of the education, the patient needs to be convinced that engaging in physical activity yields a wide multitude of positive effects in people with BC [64,75]. Although not all patients have low activity levels at the start of treatment, research has shown that only 16% of those who transition to the survivor stage are sufficiently physically active [76]. As such, in patients who are physically active at diagnosis, the emphasis of the education could be on the importance of maintaining their physical activity level throughout and after treatment. Conversely, for insufficiently active patients, the education will need to support a behavioral change towards increasing physical activity to meet the physical activity recommendations [77].

Concerning stress management, the education focuses on providing patients with a better understanding of the consequences of chronic stress and how improving stress tolerance can influence not only quality of life but also the oncological treatment outcomes [60]. This is important as advances in psychoneuroimmunology reveal a close link between stress and cancer [78]. In fact, chronic stress has been shown to speed up the development and progression of tumors, negatively impacting the clinical outcomes for cancer patients [78]. The underlying mechanism is that chronic stress activates the hypothalamic–pituitary–adrenal (HPA) axis and the sympathetic nervous system (SNS), leading to the release of stress hormones such as cortisol and catecholamines [78,79]. This prolonged neuroendocrine activation leads to inflammation, weakens immune function, and promotes conditions that support tumor growth and metastasis [78,79]. Chronic stress can lead to a continuous increase in inflammatory substances in the body, altering the immune response in a way that diminishes its tumor-fighting abilities [78,80,81]. While short-term spikes in certain inflammatory factors aid the immune system, long-term elevation results in chronic (low-grade) inflammation, which is linked to poorer cancer outcomes and various inflammatory and autoimmune conditions [78]. Besides worsening tumor growth and metastasis, chronic inflammation can also lead to cancer-related fatigue, depression, and sleep disturbances, all pertinent challenges negatively affecting the patient’s quality of life [78,82,83]. Here, it is also strongly advised to provide the education interactively while incorporating examples from the patient’s own experiences [84], such as considering their age, discussing relatable topics, or using scenarios they might encounter in their daily life. This can help to keep the patient’s attention, improve their understanding, and elicit motivation for behavioral change [84].

**Component 2.** Stress management

Being diagnosed with BC can have a notable impact on one’s psychological, social, and emotional well-being, and it has been suggested that a brief psychotherapeutic intervention with stress management can reduce psychological suffering for women with BC [7,43,85]. Considering that anxiety and/or stress is likely to be present to some extent during or following cancer treatment, such interventions can offer an opportunity to prepare patients for coping with stress and anxiety related to the BC diagnosis and treatment (i.e., surgery and subsequent cancer care) [7]. This involves teaching them stress management skills that they can apply immediately but can also be used throughout the treatment period and beyond [7].

Following stress-related education, stress management can therapeutically be approached by composing an individually tailored stress management plan together with the patient. This process can be initiated with a preliminary assessment to determine the presence and nature of stress experiences by the patient [84]. Given the subjective and individualized nature of stress [86], understanding its contribution to the patient’s daily life is crucial, as it may influence how an individual copes with the stress associated with a BC diagnosis. Subsequently, the focus can shift to the identification of personal stressors unique to the patient. Questions such as “What induces stress for you?”, “How do you typically navigate through stressful situations?”, and “How could stress potentially impact your treatment trajectory?” can provide more insight into the patient’s stress perceptions, stressors that are relevant to the patient’s life, and current stress coping strategies [84]. 

Next comes the initiation phase [84]. This is the part where different individually tailored and sustainable stress coping strategies are developed [7]. With the ECA method, a cognitive approach to better cope with stressors, patients are taught that for every single stressor, three different stress coping strategies (eliminate, change, accept) are available [48]. By presenting these coping alternatives, patients are encouraged to proactively consider how they can effectively deal with stressors as they arise in their daily lives, providing them with a sense of autonomy to choose the approach that resonates most with them [84]. Based on the individual patient, particular approaches may be recommended for further dealing with a stressor. For instance, emotional and calming coping strategies (e.g., relaxation) can be emphasized to assist someone in developing better coping mechanisms for stressors that cannot be altered or eliminated. These strategies serve as tools to navigate and accept stressors actively, turning acceptance into a proactive process that is important to successfully cope with stress. An example of the ECA method is presented in Table 1.

This process can be facilitated through the use of a Stress Reaction Record to track individual stress experiences and coping strategies applied during daily life [84]. The Stress Reaction Record serves as a valuable tool for self-reflection as patients can document stressful situations/events and describe their coping mechanisms, which encourages learning moments regarding stress coping strategies, and it can be incorporated into the ongoing development of the patient’s personalized stress management plan [84].

After the initiation phase comes the skills training, followed by confrontation [84]. In the skills training phase, patients are taught different relaxation strategies (i.e., Jacobson’s progressive relaxation therapy, visualization, and mindfulness) that can be performed at home. With the necessary skills training, patients are empowered to integrate relaxation practices seamlessly into their lives, promoting ongoing stress management. By teaching them different relaxation strategies, autonomy is given to the patient by letting them select their preferred relaxation method [84]. Table 2 briefly summarizes the different relaxation techniques that the patients learn, allowing them to select their preferred method and focus on developing proficiency in that particular technique. 

After correctly mastering the preferred relaxation technique during the skills training phase, the patient moves to the confrontation phase, applying the technique in increasingly stressful situations [84]. 

Table 3 provides an overview of a few potential cognitive-emotional barriers patients may encounter, along with examples of strategies to overcome them.

**Component 3.** Physical activity promotion

To optimally employ the teachable moment surrounding BC diagnosis [87], the exercise component of the proposed BC prehabilitation intervention follows a behavioral approach. Within this approach, overall physical activity and active lifestyle promotion is primordial. To facilitate the transition towards an active lifestyle, this component of the proposed intervention encompasses two key elements: (1) one-on-one (online) counseling guided by motivational interviewing to promote and overcome barriers to physical activity in daily life, and (2) an individually tailored home-based whole-body exercise program supported by an application providing exercise descriptions and instructional videos.

The Clinical Oncology Society of Australia (COSA) advises individuals undergoing cancer treatment to engage in 150 min of moderate-intensity physical activity or 75 min of vigorous-intensity physical activity per week, together with two strength training sessions per week [17]. This aligns with the World Health Organization’s (WHO) guidelines for physical activity in healthy individuals [6,88]. 

Hence, during the one-on-one teleconferencing sessions, patients are encouraged to achieve 150–300 min of moderate physical activity weekly [88]. This part goes hand in hand with the educational part described earlier as this physical activity goal might be difficult to achieve if the patient does not comprehend the importance of an active lifestyle. The role of the therapist in this process is to provide ongoing support to maintain the habit of an active lifestyle beyond the intervention period [89]. To facilitate a potentially necessary behavioral change and/or the maintenance of an active lifestyle, motivational interviewing principles, including feedback and positive reinforcement, can be employed throughout the one-on-one teleconferencing sessions. Moreover, allowing the patient to choose their preferred type(s) of exercise can boost motivation to adhere to a physically active lifestyle. 

Along with general physical activity promotion, part of the first one-on-one session is dedicated to the design of a home-based exercise program through shared decision making, guided by the patient’s preferences and personal goals. Telehealth offers valuable modalities to create such exercise programs providing exercise descriptions and instructional videos. Platforms with an extensive exercise database (e.g., Physitrack, MoveUP) allow for the creation of individualized exercise programs tailored to the patient needs. In line with the WHO guidelines for physical activity [88], such exercise programs should encompass approximately 30–45 min of at least moderate-intensity aerobic exercise (rated 4–6 on a 10-point perceived exertion scale), 20 min of strength training [7], and 10 min of cool-down [90]. Patients are advised to perform the exercise program at home at least twice per week [88]. 

Evidence is available that such an exercise regime is feasible and shows good adherence in presurgical patients with BC [90]. For patients who are not used to performing physical activity, this exercise program can serve as a steppingstone towards adopting a more active lifestyle. During each of the one-on-one sessions, time will be dedicated to counseling concerning the exercise program to resolve any unclarities and allow potentially necessary modifications (e.g., due to the changed patient situation early post-surgery). 

**Figure 3:**
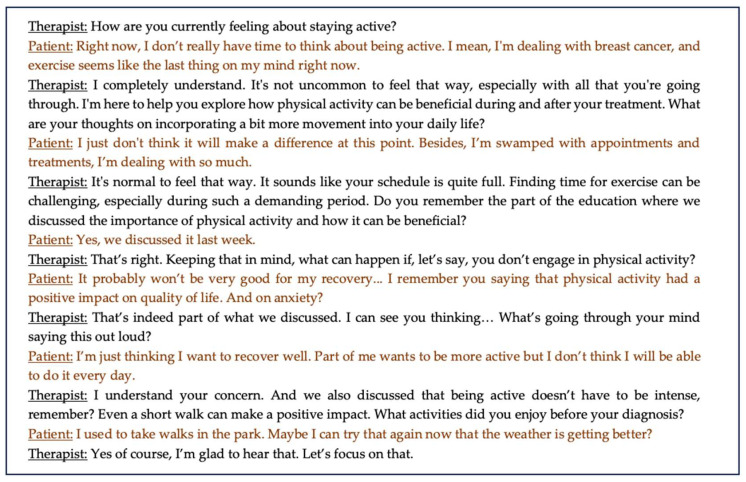
Example of a conversation between patient and therapist regarding behavioral change to initiate a more active lifestyle.

Next to understanding the importance of a physically active lifestyle, motivation also plays an important role when it comes to participating in prescribed exercise programs [89]. Hence, employing techniques that support behavioral change (e.g., goal setting, motivational interviewing, solution-focused coaching) are equally important during this component of the intervention and can play an important role in facilitating the shift towards adopting an active lifestyle (Figure 3).

Table 4 presents examples of strategies for adapting the exercise routines based on physical and emotional factors, showing how a program could be adapted to each individual patient.

#### 2.3.2. Peri-Operative Reading Material: Information Booklet

The possible psychological distress following a cancer diagnosis can influence someone’s ability to process information [7]. Therefore, the one-on-one sessions can be supplemented by an information booklet that patients can read carefully at home after their first teleprehabilitation session. The informational booklet contains a summary of the information the patients receive during the education and allows them to access essential information about prehabilitation [91]. During the second one-on-one session, the therapist can answer and explain additional questions that arose after reading the booklet. 

For patients who are proficient in computer use and prefer alternative learning methods, the informational leaflet can also be provided through more engaging formats such as video clips or an interactive app that summarizes the educational parts of BC prehabilitation. At the time of diagnosis, patients often receive numerous brochures, so presenting information in different, tech-driven formats can be refreshing and engaging. This accommodates various learning preferences and introduces a novel approach to sharing essential prehabilitation concepts.

## 3. Practical Considerations

### 3.1. Individual vs. Group Sessions

One might wonder whether BC prehabilitation could also be offered in group sessions instead of individual sessions. Group sessions offer practical advantages, fostering a sense of community and shared experience among participants, further reinforcing and stimulating each other’s behavior, and they might be perceived as more efficient from a cost perspective. However, feasibility remains a critical factor, particularly in hospital settings where the trajectories of newly diagnosed patients can vary significantly. The logistical challenges arise from the need to accommodate individualized treatment plans and diverse timelines for surgery. 

Grouping individuals with similar surgery timelines may reduce the logistical challenges and could withhold the benefits of shared experiences and support. However, it is crucial to maintain a patient-centered approach throughout prehabilitation. While group sessions offer benefits, individual sessions may be more appropriate to address the diverse needs and concerns of patients. BC diagnoses are personal, and individual sessions allow for a more tailored approach to meet specific physical, emotional, and psychological needs, which is also important to build a good therapeutic alliance [92]. In conclusion, it could be considered to apply a balance between group and individual sessions to employ the benefits of both and meet the diverse needs of BC patients. In that case, a group session could cover the more general part of the education, while the parts where individual tailoring is primordial (e.g., personalized stress management plan and the individual exercise program) are still covered in one-on-one sessions.

### 3.2. Transdisciplinary Approach 

Determining the most suitable professional to deliver BC prehabilitation sessions involves careful consideration of the varied components of education, stress management, and physical activity. While the role of a physical therapist in guiding physical activity seems intuitive, the stress management aspect raises the question of whether a physical therapist or psychologist may be the most effective care provider. The close relationship breast nurses have with patients also brings up the question of their possible involvement in delivering BC prehabilitation. Moreover, it is crucial to emphasize that the therapist administering the treatment should possess expertise in employing a behavioral approach and incorporating techniques such as motivational interviewing. Therefore, the selection of the professional delivering BC prehabilitation should not only consider their specialization but also their proficiency in implementing behavioral strategies essential for maximizing the efficacy of the intervention. Exploring a multi- or transdisciplinary approach where each expert contributes their knowledge is also a possible direction to consider [93]. However, the effectiveness of such an approach has yet to be conclusively demonstrated for BC prehabilitation, underscoring the need for further research in this area. 

Nevertheless, ensuring good transdisciplinary communication is essential to optimize the efficacy of prehabilitation. This requires that professionals involved in prehabilitation are well-informed about the medical care pathway specific to each patient. For instance, a physical therapist should have a comprehensive understanding of the patient’s medical journey to align the physical activity recommendations with the broader treatment plan. Regular communication and collaboration among the interdisciplinary team can facilitate consistency in messaging, ensuring that patients receive cohesive and accurate information across all aspects of prehabilitation. 

### 3.3. Telecommunication 

The potential of delivering prehabilitation remotely, particularly through telehealth platforms, offers numerous benefits: it reduces travel barriers, allowing patients to access services without the need for lengthy or frequent commutes; it provides flexible treatment hours, enabling patients to participate in sessions at times that align with their daily responsibilities; and it increases access to care by overcoming logistical and geographic challenges, making prehabilitation services available to a broader population [53,54,55]. However, it also introduces considerations related to accessibility and equity (e.g., the digital divide and technological barriers faced by vulnerable populations).

To address these concerns, offering in-hospital options for those with limited access to technology ensures inclusivity. Implementing user-friendly telehealth platforms, providing technical support, and offering tailored solutions allow patients to choose between remote and in-hospital sessions based on their preferences. An example of a commonly used and user-friendly telehealth platform is Physitrack (Physitrack Limited, London, UK) [94]. Physitrack is an online platform accessible via both a web page and an application called PhysiApp. It includes an extensive library of exercise programs with instructional videos, video call capabilities for real-time therapist–patient interaction, and tracking and logbook features that allow both parties to monitor progress and adjust treatment plans as needed. Communication tools, such as chat functions and secure messaging, facilitate continuous support by enabling patients to seek clarifications or report concerns [94]. Such communication functions can also be highly beneficial as a support system for home exercises. It entails enabling patients to reach out for assistance or clarification regarding their home exercises through a chat function or a dedicated telephone helpline. Therapists can be contacted through this channel, and dedicated response protocols (e.g., replies within 24 h) can be established to enhance the accessibility and effectiveness of remote support, but also for better patient engagement and satisfaction.

However, despite its benefits, telehealth also presents challenges, particularly regarding accessibility and equity. Vulnerable populations may face technological barriers, including limited access to devices, unreliable internet connections, or insufficient digital literacy. To address these issues, healthcare providers should offer in-hospital alternatives for patients who lack access to technology and ensure inclusivity by providing technical support, such as tutorials and troubleshooting assistance. Proactive measures, including the provision of devices or internet access assistance, can further bridge the digital divide and expand access to care. Ultimately, telehealth should complement rather than replace in-person care.

### 3.4. Time Frame 

The challenging time frame between diagnosis and first-line treatment presents a significant logistical hurdle for implementing prehabilitation. The current structure of two pre-surgical and two post-surgical sessions may face constraints if the pre-operative period becomes shorter in the future. This potential reduction in the pre-operative period necessitates a flexible and adaptive approach to BC prehabilitation. 

Strategies may include optimizing the program’s efficiency, incorporating digital platforms, prioritizing essential components, and maintaining open communication among healthcare providers to meet the evolving needs of patients in a rapidly changing medical field. For instance, essential components could be prioritized for patients with limited time before surgery. This would involve identifying key components that can be delivered efficiently and have the most significant impact on patient outcomes within a compressed timeframe.

## 4. Conclusions

BC prehabilitation emerges as a valuable and multifaceted intervention that addresses the physical, psychological, and educational needs of patients facing BC diagnosis and treatment. By integrating education, stress management, and physical activity promotion, the proposed approach aims to bridge the gap between diagnosis and oncological treatment and provides a holistic preparation for surgery and postoperative rehabilitation in breast cancer patients.

By delivering prehabilitation remotely (i.e., teleprehabilitation), patients can value multiple benefits such as reduced travel barriers and flexible treatment hours. In addition to its accessibility and interactive features, e-Health also offers the opportunity to enhance the educational aspect of prehabilitation. Implementing platforms with essential features (e.g., exercise libraries with instructional videos, video call capabilities, tracking features, communication tools, feedback mechanisms) and proactive measures, such as providing devices or internet access assistance, can help bridge the digital divide and ensure equitable access to the benefits of prehabilitation for all patient populations.

Practical considerations, such as the optimal delivery format, timing, and the choice of involved care professionals, highlight the importance of flexibility and adaptability to meet the evolving needs of BC patients. Ultimately, BC prehabilitation represents a holistic approach that not only prepares patients for surgery but also empowers them to actively engage in their care, promoting a comprehensive and patient-centric approach to BC treatment.

Further research on breast cancer prehabilitation should explore the long-term impact of teleprehabilitation on patient outcomes, including physical, psychological, and quality-of-life measurements, as well as its cost-effectiveness, as these aspects could provide robust evidence to integrate teleprehabilitation into standard care. Additionally, the potential of artificial intelligence and machine learning to customize teleprehabilitation programs offers a promising avenue for innovation, allowing tailored interventions based on patient-specific needs. Such advancements could optimize patient engagement and outcomes while improving the efficiency of healthcare delivery systems.

## Figures and Tables

**Table 1:** Example of the eliminate–change–accept (ECA) method [48].

Eliminate	Change	Accept
A patient with breast cancer is experiencing stress related to returning to work. First, explore whether the stressor can be eliminated. For stress about returning to work, the patient might consider extended leave. Is it possible to arrange an extended medical leave to allow more time for recovery and thus eliminate the immediate stress of returning to work too soon? If the stressor is more related to commuting to work or being in a demanding work environment for instance, another way of eliminating this stressor could be to negotiate with their employer to work from home if their job allows it.	If eliminating the stressor is not feasible, the next step would be to see if it can be changed. Is it possible to discuss a phased return-to-work plan with their employer (e.g., part-time hours or lighter duties) or to request reasonable adjustments at work (e.g., more flexible working hours, frequent breaks to manage fatigue)?	If the stressor cannot be eliminated or changed, acceptance is the final step. Acceptance involves acknowledging the stressor and finding ways to live with it. This does not mean they have to face their misery alone. In fact, the therapist is there to help them accept the situation. By thoroughly exploring all options, the patient becomes aware of the circumstances, which will aid in their acceptance. An idea could be to teach them relaxation skills to help them manage the related stress.

**Table 2:** Overview of the relaxation techniques.

Technique	Example
Jacobson’s progressive relaxation therapy	This technique focuses on the deliberatie contraction and relaxation of different muscle groups in the body, usually starting from the feet and working upward. The process involves tightening a muscle group (e.g., clenching you first) for about 5–10 s, then releasing the tension and noticing the sensation of relaxation for 15–20 s before moving on to the next muscle group.
Visualization	In this technique, you imagine yourself in a calming setting, like a peaceful beach or serene forest, engaging all your senses to make the scene as vivid as possible. This helps distract your mind from stress and fosters a sense of tranquility.
Mindfulness	Mindfulness is a practice of focusing your attention on the present moment, often by observing your breath, sensations, or surroundings. It helps reduce stress by grounding you in the here and now. Mindful breathing is an example where you focus on your breath as it flows in and out, holding your attention to the sensation of your breath, noticing the rhythm, temperature, and how your chest and belly moves with each inhale and exhale. Another common example is the full body scan. This mindfulness technique involves directing attention to each part of the body, beginning at the toes and moving upward, to observe sensations and consciously release tension. Contrary to Jacobson’s progressive relaxation therapy, in this body scan, you focus on passively observing sensations in each part of the body without trying to change them.

**Table 3:** Examples of barriers to stress management.

Barrier	Strategy
**Mistrust in psychosocial interventions** Patients may undervalue psychosocial approaches, viewing them as less important or as “not for them”	Tailor the stress-reduction activities to align with their preferences. Jacobson’s progressive muscle relaxation, for example, bridges the gap between physical and mental relaxation by providing a tangible, action-oriented approach that feels grounded and practical, which can be interesting for patients who are skeptical about psychosocial interventions. Communication strategy: normalize and explore their concerns, challenge their perceptions/beliefs, share examples of patients who have benefited from these tools in a way that feels relatable, etc.
**Lack of time or energy** Patients undergo diagnostic tests, treatments, or are preparing for surgery, which might make them feel like they have no time or energy for stress-reduction activities	Start with brief techniques (5–10 min of breathing or mindfulness exercises that can be done while resting) and focus on how these techniques could be integrated into the patient’s routine. Communication strategy: normalize and explore their concerns, validate their emotions, help them identify small and manageable steps to implement the stress-reduction activities into their daily lives, etc.
**Cultural or personal beliefs** Patients might have cultural or personal views that stigmatize seeking help for stress, perceiving it as a sign of weakness	Offer stress-reduction techniques aligned with the patient’s values (e.g., prayer, family involvement). Frame stress management as a way to build strength and resilience for the challenges ahead. Communication strategy: respect their (cultural) values.

**Table 4:** Examples of strategies for adapting the program based on physical and emotional factors.

Category		Strategy
Based on physical activity level	Beginners/low fitness level	Incorporate gentle, low-impact exercises to build confidence and stamina. Start with short, feasible sessions and progress over time. Shift the focus to maintaining consistency, rather than intensity or performance.
	High fitness level	Maintain physical fitness with more intense, varied exercises while ensuring safety and avoiding overexertion. Normalize the experience of fatigue and reassure patients that this is expected during treatment: use a flexible schedule (e.g., adjusting sessions based on daily energy) and encourage shorter, lighter sessions when energy is low.
Based on cognitive/emotional barriers	Low motivation or fear	Focus on building trust and confidence. Tailor programs to the patient’s current capacity and incorporate activities that the patient enjoys. Identify patient-specific goals and break them up into small subgoals that are achievable in the short term. Celebrate small achievements. Communication: normalize and explore their concerns, challenge their perceptions/beliefs, etc.

## References

[1] Bray F., Laversanne M., Sung H., Ferlay J., Siegel R.L., Soerjomataram I., Jemal A. (2024). Global cancer statistics 2022: GLOBOCAN estimates of incidence and mortality worldwide for 36 cancers in 185 countries. CA Cancer J Clin.

[2] Belgian Cancer Registry (2023). Cancer Fact Sheets Incidence Year 2021.

[3] Martínez Arroyo O., Andreu Vaíllo Y., Martínez López P., Galdón Garrido M.J. (2019). Emotional distress and unmet supportive care needs in survivors of breast cancer beyond the end of primary treatment. Support. Care Cancer.

[4] Vuksanovic D., Sanmugarajah J., Lunn D., Sawhney R., Eu K., Liang R. (2021). Unmet needs in breast cancer survivors are common, and multidisciplinary care is underutilised: The Survivorship Needs Assessment Project. Breast Cancer.

[5] Howe L., Husband A., Robinson-Barella A. (2024). Prescribing pre- and post-operative physical activity interventions for people undergoing breast cancer surgery: A qualitative systematic review. Cancer Med..

[6] Toohey K., Hunter M., McKinnon K., Casey T., Turner M., Taylor S., Paterson C. (2023). A systematic review of multimodal prehabilitation in breast cancer. Breast Cancer Res. Treat..

[7] Santa Mina D., Brahmbhatt P., Lopez C., Baima J., Gillis C., Trachtenberg L., Silver J.K. (2017). The Case for Prehabilitation Prior to Breast Cancer Treatment. PM&R.

[8] Van Beek F.E., Wijnhoven L.M., Holtmaat K., Custers J.A., Prins J.B., Verdonck-de Leeuw I.M., Jansen F. (2021). Psychological problems among cancer patients in relation to healthcare and societal costs: A systematic review. Psycho-Oncol..

[9] UK C.R. (2021). Breast Cancer Statistics. https://www.cancerresearchuk.org/health-professional/cancer-statistics/statistics-by-cancer-type/breast-cancer#heading-Six.

[10] Wu F., Laza-Cagigas R., Pagarkar A., Olaoke A., El Gammal M., Rampal T. (2021). The feasibility of prehabilitation as part of the breast cancer treatment pathway. PM&R.

[11] Miller K.D., Siegel R.L., Lin C.C., Mariotto A.B., Kramer J.L., Rowland J.H., Stein K.D., Alteri R., Jemal A. (2016). Cancer treatment and survivorship statistics, 2016. CA A Cancer J. Clin..

[12] de Jesus Leite M.A.F., Puga G.M., Arantes F.J., Oliveira C.J.F., Cunha L.M., Bortolini M.J.S., Penha-Silva N. (2018). Effects of combined and resistance training on the inflammatory profile in breast cancer survivors: A systematic review. Complement. Ther. Med..

[13] Mishra S.I., Scherer R.W., Geigle P.M., Berlanstein D.R., Topaloglu O., Gotay C.C., Snyder C. (2012). Exercise interventions on health-related quality of life for cancer survivors. Cochrane Database Syst. Rev..

[14] Cheng K.K.F., Lim Y.T.E., Koh Z.M., San Tam W.W. (2017). Home-based multidimensional survivorship programmes for breast cancer survivors. Cochrane Database Syst. Rev..

[15] National Institute for Health and Care Excellence (NICE) (2024). Early and Locally Advanced Breast Cancer: Diagnosis and Management.

[16] Koninklijk Nederlands Genootschap voor Fysiotherapie (KNGF) (2022). KNGF-Richtlijn Oncologie.

[17] Cormie P., Atkinson M., Bucci L., Cust A., Eakin E., Hayes S., McCarthy S., Murnane A., Patchell S., Adams D. (2018). Clinical Oncology Society of Australia position statement on exercise in cancer care. Med. J. Aust..

[18] Zagalaz-Anula N., Mora-Rubio M.J., Obrero-Gaitán E., Del-Pino-Casado R. (2022). Recreational physical activity reduces breast cancer recurrence in female survivors of breast cancer: A meta-analysis. Eur. J. Oncol. Nurs..

[19] Elshahat S., Treanor C., Donnelly M. (2021). Factors influencing physical activity participation among people living with or beyond cancer: A systematic scoping review. Int. J. Behav. Nutr. Phys. Act..

[20] Orange S.T., Northgraves M.J., Marshall P., Madden L.A., Vince R.V. (2018). Exercise prehabilitation in elective intra-cavity surgery: A role within the ERAS pathway? A narrative review. Int. J. Surg..

[21] Silver J.K., Baima J. (2013). Cancer prehabilitation: An opportunity to decrease treatment-related morbidity, increase cancer treatment options, and improve physical and psychological health outcomes. Am. J. Phys. Med. Rehabil..

[22] Scheede-Bergdahl C., Minnella E., Carli F. (2019). Multi-modal prehabilitation: Addressing the why, when, what, how, who and where next?. Anaesthesia.

[23] Brahmbhatt P., Sabiston C.M., Lopez C., Chang E., Goodman J., Jones J., McCready D., Randall I., Rotstein S., Santa Mina D. (2020). Feasibility of prehabilitation prior to breast cancer surgery: A mixed-methods study. Front. Oncol..

[24] Rossi C., Maggiore C., Rossi M.M., Filippone A., Guarino D., Di Micco A., Forcina L., Magno S. (2021). A Model of an Integrative Approach to Breast Cancer Patients. Integr. Cancer Ther..

[25] Chou Y.-J., Kuo H.-J., Shun S.-C. (2018). Cancer Prehabilitation Programs and Their Effects on Quality of Life. Oncol. Nurs. Forum.

[26] Wiener A.A., Hanlon B.M., Schumacher J.R., Vande Walle K.A., Wilke L.G., Neuman H.B. (2023). Reexamining Time From Breast Cancer Diagnosis to Primary Breast Surgery. JAMA Surg..

[27] Eaglehouse Y.L., Georg M.W., Shriver C.D., Zhu K. (2019). Time-to-surgery and overall survival after breast cancer diagnosis in a universal health system. Breast Cancer Res. Treat..

[28] Desborough J. (2000). The stress response to trauma and surgery. Br. J. Anaesth..

[29] Khoo B., Boshier P.R., Freethy A., Tharakan G., Saeed S., Hill N., Williams E.L., Moorthy K., Tolley N., Jiao L.R. (2017). Redefining the stress cortisol response to surgery. Clin. Endocrinol..

[30] Soares Falcetta F., de Araújo Vianna Träsel H., de Almeida F.K., Rangel Ribeiro Falcetta M., Falavigna M., Dornelles Rosa D. (2018). Effects of physical exercise after treatment of early breast cancer: Systematic review and meta-analysis. Breast Cancer Res. Treat..

[31] Patterson R.E., Cadmus L.A., Emond J.A., Pierce J.P. (2010). Physical activity, diet, adiposity and female breast cancer prognosis: A review of the epidemiologic literature. Maturitas.

[32] Kim J., Choi W.J., Jeong S.H. (2013). The effects of physical activity on breast cancer survivors after diagnosis. J. Cancer Prev..

[33] Kokts-Porietis R.L., Stone C.R., Friedenreich C.M., Froese A., McDonough M., McNeil J. (2019). Breast cancer survivors’ perspectives on a home-based physical activity intervention utilizing wearable technology. Support. Care Cancer.

[34] Agasi-Idenburg C.S., Koning-van Zuilen M., Westerman M.J., Punt C.J., Aaronson N.K., Stuiver M.M. (2020). “I am busy surviving”-Views about physical exercise in older adults scheduled for colorectal cancer surgery. J. Geriatr. Oncol..

[35] Banerjee S., Semper K., Skarparis K., Naisby J., Lewis L., Cucato G., Mills R., Rochester M., Saxton J. (2021). Patient perspectives of vigorous intensity aerobic interval exercise prehabilitation prior to radical cystectomy: A qualitative focus group study. Disabil. Rehabil..

[36] Horn-Hofmann C., Scheel J., Dimova V., Parthum A., Carbon R., Griessinger N., Sittl R., Lautenbacher S. (2018). Prediction of persistent post-operative pain: Pain-specific psychological variables compared with acute post-operative pain and general psychological variables. Eur. J. Pain.

[37] Masselin-Dubois A., Attal N., Fletcher D., Jayr C., Albi A., Fermanian J., Bouhassira D., Baudic S. (2013). Are psychological predictors of chronic postsurgical pain dependent on the surgical model? A comparison of total knee arthroplasty and breast surgery for cancer. J. Pain.

[38] Alodaibi F.A., Minick K.I., Fritz J.M. (2013). Do preoperative fear avoidance model factors predict outcomes after lumbar disc herniation surgery? A systematic review. Chiropr. Man. Ther..

[39] Sanford N.N., Sher D.J., Butler S.S., Xu X., Ahn C., Aizer A.A., Mahal B.A. (2019). Prevalence of chronic pain among cancer survivors in the United States, 2010–2017. Cancer.

[40] Schreiber K.L., Kehlet H., Belfer I., Edwards R.R. (2014). Predicting, preventing and managing persistent pain after breast cancer surgery: The importance of psychosocial factors. Pain Manag..

[41] Schreiber K.L., Zinboonyahgoon N., Xu X., Spivey T., King T., Dominici L., Partridge A., Golshan M., Strichartz G., Edwards R.R. (2019). Preoperative psychosocial and psychophysical phenotypes as predictors of acute pain outcomes after breast surgery. J. Pain.

[42] Meretoja T.J., Leidenius M.H., Tasmuth T., Sipilä R., Kalso E. (2014). Pain at 12 months after surgery for breast cancer. JAMA.

[43] Tsimopoulou I., Pasquali S., Howard R., Desai A., Gourevitch D., Tolosa I., Vohra R. (2015). Psychological prehabilitation before cancer surgery: A systematic review. Ann. Surg. Oncol..

[44] Brahmbhatt P., Look Hong N.J., Sriskandarajah A., Alavi N., Selvadurai S., Berger-Richardson D., Lemon-Wong S., Mascarenhas J., Gibson L., Rapier T. (2024). A Feasibility Randomized Controlled Trial of Prehabilitation During Neoadjuvant Chemotherapy for Women with Breast Cancer: A Mixed Methods Study. Ann. Surg. Oncol..

[45] Garssen B., Boomsma M.F., Meezenbroek Ede J., Porsild T., Berkhof J., Berbee M., Visser A., Meijer S., Beelen R.H. (2013). Stress management training for breast cancer surgery patients. Psychooncology.

[46] Miller W.R. (1996). Motivational interviewing: Research, practice, and puzzles. Addict. Behav..

[47] Miller W.R., Rollnick S. (2009). Ten things that motivational interviewing is not. Behav. Cogn. Psychother..

[48] Nijs J., Roose E., Lahousse A., Mostaqim K., Reynebeau I., De Couck M., Beckwee D., Huysmans E., Bults R., van Wilgen P. (2021). Pain and Opioid Use in Cancer Survivors: A Practical Guide to Account for Perceived Injustice. Pain Physician.

[49] Nijs J., Wijma A.J., Willaert W., Huysmans E., Mintken P., Smeets R., Goossens M., van Wilgen C.P., Van Bogaert W., Louw A. (2020). Integrating motivational interviewing in pain neuroscience education for people with chronic pain: A practical guide for clinicians. Phys. Ther..

[50] van der Kloot W.A., Uchida Y., Inoue K., Kobayashi K., Yamaoka K., Nortier H., Kaptein A.A. (2016). The effects of illness beliefs and chemotherapy impact on quality of life in Japanese and Dutch patients with breast or lung cancer. Chin. Clin. Oncol..

[51] Ferreira V., Agnihotram R.V., Bergdahl A., van Rooijen S.J., Awasthi R., Carli F., Scheede-Bergdahl C. (2018). Maximizing patient adherence to prehabilitation: What do the patients say?. Support. Care Cancer.

[52] Parker N.H., Lee R.E., O’Connor D.P., Ngo-Huang A., Petzel M.Q., Schadler K., Wang X., Xiao L., Fogelman D., Simpson R. (2019). Supports and barriers to home-based physical activity during preoperative treatment of pancreatic cancer: A mixed-methods study. J. Phys. Act. Health.

[53] Zimmerman B.S., Seidman D., Berger N., Cascetta K.P., Nezolosky M., Trlica K., Ryncarz A., Keeton C., Moshier E., Tiersten A. (2020). Patient perception of telehealth services for breast and gynecologic oncology care during the COVID-19 pandemic: A single center survey-based study. J. Breast Cancer.

[54] Van Egmond M., Van Der Schaaf M., Vredeveld T., Vollenbroek-Hutten M., van Berge Henegouwen M., Klinkenbijl J., Engelbert R. (2018). Effectiveness of physiotherapy with telerehabilitation in surgical patients: A systematic review and meta-analysis. Physiotherapy.

[55] Bartolo A., Pacheco E., Rodrigues F., Pereira A., Monteiro S., Santos I.M. (2019). Effectiveness of psycho-educational interventions with telecommunication technologies on emotional distress and quality of life of adult cancer patients: A systematic review. Disabil. Rehabil..

[56] Wu F., Rotimi O., Laza-Cagigas R., Rampal T. (2021). The Feasibility and Effects of a Telehealth-Delivered Home-Based Prehabilitation Program for Cancer Patients during the Pandemic. Curr. Oncol..

[57] Knoerl R., Giobbie-Hurder A., Sannes T.S., Chagpar A.B., Dillon D., Dominici L.S., Frank E.S., Golshan M., McTiernan A., Rhei E. (2022). Exploring the impact of exercise and mind–body prehabilitation interventions on physical and psychological outcomes in women undergoing breast cancer surgery. Support. Care Cancer.

[58] Lee H., Wiggers J., Kamper S.J., Williams A., O’Brien K.M., Hodder R.K., Wolfenden L., Yoong S.L., Campbell E., Haskins R. (2017). Mechanism evaluation of a lifestyle intervention for patients with musculoskeletal pain who are overweight or obese: Protocol for a causal mediation analysis. BMJ Open.

[59] Testa A., Iannace C., Di Libero L., Caracciolo F. (2013). Strengths of early physical rehabilitation programs in surgical breast cancer patients: Results of a randomized control study. BMC Proc..

[60] Hanalis-Miller T., Nudelman G., Ben-Eliyahu S., Jacoby R. (2022). The effect of pre-operative psychological interventions on psychological, physiological, and immunological indices in oncology patients: A scoping review. Front. Psychol..

[61] Ghavami H., Akyolcu N. (2017). The impact of lifestyle interventions in breast cancer women after completion of primary therapy: A randomized study. J. Breast Health.

[62] Pistelli M., Natalucci V., Scortichini L., Agostinelli V., Lenci E., Crocetti S., Merloni F., Bastianelli L., Taus M., Fumelli D. (2021). The impact of lifestyle interventions in high-risk early breast cancer patients: A modeling approach from a single institution experience. Cancers.

[63] Hwang E.S., Nho J.-H. (2019). Lifestyle intervention for breast cancer women. J. Lifestyle Med..

[64] Ortega M.A., Fraile-Martínez O., García-Montero C., Pekarek L., Guijarro L.G., Castellanos A.J., Sanchez-Trujillo L., García-Honduvilla N., Álvarez-Mon M., Buján J. (2020). Physical activity as an imperative support in breast cancer management. Cancers.

[65] Lahart I.M., Metsios G.S., Nevill A.M., Carmichael A.R. (2015). Physical activity, risk of death and recurrence in breast cancer survivors: A systematic review and meta-analysis of epidemiological studies. Acta Oncol..

[66] Heitz A.E., Baumgartner R.N., Baumgartner K.B., Boone S.D. (2018). Healthy lifestyle impact on breast cancer-specific and all-cause mortality. Breast Cancer Res. Treat..

[67] Chen L.H., Irwin M.R., Olmstead R., Haque R. (2022). Association of Physical Activity With Risk of Mortality Among Breast Cancer Survivors. JAMA Netw. Open.

[68] Friedenreich C.M., Gregory J., Kopciuk K.A., Mackey J.R., Courneya K.S. (2009). Prospective cohort study of lifetime physical activity and breast cancer survival. Int. J. Cancer.

[69] Møller T., Andersen C., Lillelund C., Bloomquist K., Christensen K.B., Ejlertsen B., Tuxen M., Oturai P., Breitenstein U., Kolind C. (2020). Physical deterioration and adaptive recovery in physically inactive breast cancer patients during adjuvant chemotherapy: A randomised controlled trial. Sci. Rep..

[70] Servaes P., Verhagen C., Bleijenberg G. (2002). Fatigue in cancer patients during and after treatment: Prevalence, correlates and interventions. Eur. J. Cancer.

[71] Goedendorp M., Gielissen M., Verhagen C., Peters M., Bleijenberg G. (2008). Severe fatigue and related factors in cancer patients before the initiation of treatment. Br. J. Cancer.

[72] Lipsett A., Barrett S., Haruna F., Mustian K., O’Donovan A. (2017). The impact of exercise during adjuvant radiotherapy for breast cancer on fatigue and quality of life: A systematic review and meta-analysis. Breast.

[73] Zanghì M., Petrigna L., Maugeri G., D’Agata V., Musumeci G. (2022). The practice of physical activity on psychological, mental, physical, and social wellbeing for breast-cancer survivors: An umbrella review. Int. J. Environ. Res. Public Health.

[74] Hermelink R., Leitzmann M.F., Markozannes G., Tsilidis K., Pukrop T., Berger F., Baurecht H., Jochem C. (2022). Sedentary behavior and cancer–an umbrella review and meta-analysis. Eur. J. Epidemiol..

[75] Schmid D., Leitzmann M. (2014). Association between physical activity and mortality among breast cancer and colorectal cancer survivors: A systematic review and meta-analysis. Ann. Oncol..

[76] Boyle T., Vallance J.K., Ransom E.K., Lynch B.M. (2016). How sedentary and physically active are breast cancer survivors, and which population subgroups have higher or lower levels of these behaviors?. Support. Care Cancer.

[77] World Health Organization (WHO) (2020). WHO Guidelines on Physical Activity and Sedentary Behaviour.

[78] Liu Y., Tian S., Ning B., Huang T., Li Y., Wei Y. (2022). Stress and cancer: The mechanisms of immune dysregulation and management. Front. Immunol..

[79] Zhang L., Pan J., Chen W., Jiang J., Huang J. (2020). Chronic stress-induced immune dysregulation in cancer: Implications for initiation, progression, metastasis, and treatment. Am. J. Cancer Res..

[80] Gouin J.-P., Glaser R., Malarkey W.B., Beversdorf D., Kiecolt-Glaser J. (2012). Chronic stress, daily stressors, and circulating inflammatory markers. Health Psychol..

[81] Elenkov I.J., Chrousos G.P. (2002). Stress hormones, proinflammatory and antiinflammatory cytokines, and autoimmunity. Ann. New York Acad. Sci..

[82] Bower J.E., Lamkin D.M. (2013). Inflammation and cancer-related fatigue: Mechanisms, contributing factors, and treatment implications. Brain Behav. Immun..

[83] Irwin M.R. (2013). Depression and insomnia in cancer: Prevalence, risk factors, and effects on cancer outcomes. Curr. Psychiatry Rep..

[84] Willaert W., Leysen L., Lenoir D., Meeus M., Cagnie B., Nijs J., Sterling M., Coppieters I. (2021). Combining stress management with pain neuroscience education and exercise therapy in people with whiplash-associated disorders: A clinical perspective. Phys. Ther..

[85] Tang M., Liu X., Wu Q., Shi Y. (2020). The effects of cognitive-behavioral stress management for breast cancer patients: A systematic review and meta-analysis of randomized controlled trials. Cancer Nurs..

[86] Wijma A.J., van Wilgen C.P., Meeus M., Nijs J. (2016). Clinical biopsychosocial physiotherapy assessment of patients with chronic pain: The first step in pain neuroscience education. Physiother. Theory Pract..

[87] Demark-Wahnefried W., Aziz N.M., Rowland J.H., Pinto B.M. (2005). Riding the crest of the teachable moment: Promoting long-term health after the diagnosis of cancer. J. Clin. Oncol..

[88] Bull F.C., Al-Ansari S.S., Biddle S., Borodulin K., Buman M.P., Cardon G., Carty C., Chaput J.-P., Chastin S., Chou R. (2020). World Health Organization 2020 guidelines on physical activity and sedentary behaviour. Br. J. Sports Med..

[89] Teo J.L., Zheng Z., Bird S.R. (2022). Identifying the factors affecting ‘patient engagement’in exercise rehabilitation. BMC Sports Sci. Med. Rehabil..

[90] Ligibel J.A., Dillon D., Giobbie-Hurder A., McTiernan A., Frank E., Cornwell M., Pun M., Campbell N., Dowling R.J., Chang M.C. (2019). Impact of a pre-operative exercise intervention on breast cancer proliferation and gene expression: Results from the pre-operative health and body (PreHAB) study. Clin. Cancer Res..

[91] Ibrahim M., Lau G.J., Smirnow N., Buono A.T., Cooke A., Gartshore K., Loiselle C.G., Johnson K. (2018). A multidisciplinary preoperative teaching session for women awaiting breast cancer surgery: A quality improvement initiative. Rehabil. Process Outcome.

[92] Kinney M., Seider J., Beaty A.F., Coughlin K., Dyal M., Clewley D. (2020). The impact of therapeutic alliance in physical therapy for chronic musculoskeletal pain: A systematic review of the literature. Physiother. Theory Pract..

[93] Wijma A.J., Speksnijder C.M., Crom-Ottens A.F., Knulst-Verlaan J.C., Keizer D., Nijs J., van Wilgen C.P. (2018). What is important in transdisciplinary pain neuroscience education? A qualitative study. Disabil. Rehabil..

[94] Physitrack. https://www.physitrack.com/?lang=nl.

